# Supplementing clinical lactation studies with PBPK modeling to inform drug therapy in lactating mothers: Prediction of primaquine exposure as a case example

**DOI:** 10.1002/psp4.13090

**Published:** 2023-12-12

**Authors:** Xian Pan, Khaled Abduljalil, Lisa M. Almond, Amita Pansari, Karen Rowland Yeo

**Affiliations:** ^1^ Certara UK Limited (Simcyp Division) Sheffield UK

## Abstract

Evaluating the safety of primaquine (PQ) during breastfeeding requires an understanding of its pharmacokinetics (PKs) in breast milk and its exposure in the breastfed infant. Physiologically‐based PK (PBPK) modeling is primed to assess the complex interplay of factors affecting the exposure of PQ in both the mother and the nursing infant. A published PBPK model for PQ describing the metabolism by monoamine oxidase A (MAO‐A; 90% contribution) and cytochrome P450 2D6 (CYP2D6; 10%) in adults was applied to predict the exposure of PQ in mothers and their breastfeeding infants. Plasma exposures following oral daily dosing of 0.5 mg/kg in the nursing mothers in a clinical lactation study were accurately captured, including the observed ranges. Reported infant daily doses based on milk data from the clinical study were used to predict the exposure of PQ in breastfeeding infants greater than or equal to 28 days. On average, the predicted exposures were less than or equal to 0.13% of the mothers. Furthermore, in simulations involving neonates less than 28 days, PQ exposures remain less than 0.16% of the mothers. Assuming that MAO‐A increases slowly with age, the predicted relative exposure of PQ remains low in neonates (<0.46%). Thus, the findings of our study support the recommendation made by the authors who reported the results of the clinical lactation study, that is, that when put into context of safety data currently available in children, PQ should not be withheld in lactating women as it is unlikely to cause adverse events in breastfeeding infants greater than or equal to 28 days old.

**Study Highlights**

**WHAT IS THE CURRENT KNOWLEDGE ON THIS TOPIC?**

Physiologically‐based pharmacokinetic (PBPK) models can predict exposures in infants and neonates accounting for age‐related changes in physiology. Therefore, this approach is well‐positioned to support medicine use in mothers by characterizing drug exposure in breastfeeding infants

**WHAT QUESTION DID THIS STUDY ADDRESS?**

How PBPK models can be used to supplement available clinical lactation data on primaquine (PQ) by providing more comprehensive information regarding infant drug exposures through breastfeeding

**WHAT DOES THIS STUDY ADD TO OUR KNOWLEDGE?**

A published PBPK model for PQ accurately captured the observed concentration‐time profiles in mothers participating in the clinical lactation study. Thereafter, the model confirmed that exposures in breastfeeding infants are at least 100‐fold lower than in the mothers. Simulations indicated this was also the case in neonates for whom there are no clinical data

**HOW MIGHT THIS CHANGE DRUG DISCOVERY, DEVELOPMENT, AND/OR THERAPEUTICS?**

This example demonstrates application of the use of PBPK modeling to supplement clinical lactation data and inform decision making on PQ for use during breastfeeding.

## INTRODUCTION

Primaquine (PQ) is an 8‐aminoquinoline used for the treatment of *Plasmodium vivax* and *Plasmodium ovale* malaria.^1^ One of the biggest challenges associated with the treatment of *P. vivax* infection is how to prevent relapses, especially as dormant liver‐stage parasites (hypnozoites) can be re‐activated, weeks or even years after the initial onset of illness.^2^ Clinical symptoms of *P. vivax* infection include jaundice, severe anemia, multi‐organ failure, and thrombocytopenia. In endemic areas, children often also present with chronic malnutrition,^3^ and during pregnancy, if the mothers contract the disease, this can result in spontaneous abortion, premature deliveries, or low birth weight newborns.^4^ Despite the global burden of *P. vivax* being significantly reduced between the years of 2010 and 2019, control and elimination of the infection remains a problem because of the relapses caused by activation of the hypnozoites.^5^ As PQ is the only currently approved drug that can treat the liver stage of the parasite, the World Health Organization (WHO) recommends standard antimalarial medication followed by daily dosing of 0.25 mg/kg PQ for 14 days in adults.^6^ One of the main side effects of PQ is that it may cause hemolysis, a condition which could potentially be life‐threatening, especially in patients with glucose‐6‐phosphate dehydrogenase (G6PD) deficiency.^7^

Currently, the WHO treatment guidelines recommend delaying PQ administration in lactating women by 6 months until breastfeeding infants are determined to be normal for G6PD.^6^ The US Food and Drug Administration (FDA) drug label states that in lactating mothers, either nursing or drug administration should be discontinued but that the decision needs to be weighed against the importance of the drug to the mother.^8^ Generally, guidance on PQ administration in lactating women appears to be based on the risk of hemolysis occurring in nursing infants. In a recent clinical study, PQ pharmacokinetic (PK) data were obtained in healthy G6PD‐normal women with previous *P. vivax* infection and their healthy G6PD‐normal breastfeeding infants.^9^ In 20 mother‐infant pairs, PQ plasma concentrations in infants were below measurement thresholds in all but one plasma sample. Concentrations of PQ in breast milk were reported to be very low and the relative infant daily dose (RIDD) was estimated to be ~0.6% of that of the mother. This is significantly lower than the threshold set by the WHO Working Group who proposed that drugs with an RIDD greater than 10% may not be safe in infants.^10^ Furthermore, no differences in absolute hematocrit between infants enrolled in this study and age‐matched infants from the local population were reported and biochemical analyses supported a lack of evidence of PQ toxicity. Based on these data, the authors concluded that PQ should not be withheld in lactating women as it was unlikely to cause adverse events in breastfeeding infants greater than or equal to 28 days old.^9^ Neonates (<28 days old) were excluded from the clinical study and hence no conclusions could be drawn for this age group.

Physiologically based PK (PBPK) models, which accommodate the complex interplay between drug‐related characteristics and physiological parameters in the populations of interest, present a mechanistic approach to predict the PK of drugs in nursing mothers^11^ and breastfeeding infants.^11^, ^12^, ^13^ Pediatric PBPK models which account for age‐related changes in organ/tissue development, blood flows, enzymes and transporters are primed for determining the exposure of a drug in neonates and infants.^14^, ^15^ The development and verification of a PBPK model for PQ describing the metabolism by monoamine oxidase A (MAO‐A) and cytochrome P450 2D6 (CYP2D6) was reported previously.^16^ The aim of this study was to use our recently published PBPK model for PQ to bridge knowledge gaps (that could not be addressed clinically) by predicting the exposure of the drug in mothers and their breastfeeding infants, including neonates less than 28 days old. Specifically, infant daily doses (IDDs) estimated from the PQ milk exposure in mothers were used in simulations of virtual infants with time‐varying physiology, including relevant ontogenies. Thereafter, various simulations were run to address the uncertainty associated with key factors affecting the RIDD/infant exposures in the actual clinical study. As the published PQ model was developed in adults, simulations were also conducted to verify the performance of the model in children using available clinical data.

## METHODS

### Software

The Simcyp (version 21.1) population‐based PBPK simulator (Simcyp, Sheffield, UK) was used to generate plasma concentration‐time profiles of PQ in nursing mothers and infants. The PBPK model developed previously for PQ, included MAO‐A‐ and CYP2D6‐mediated metabolism; relative contributions of each of the enzymes in adults were 90% and 10%, respectively.^16^ Clinical study data from the literature were digitized with GetData Graph Digitizer version 2.22.

### Clinical data

In the published clinical study,^9^ 20 G6PD‐normal mothers (18–40 years), with a history of *P. vivax* infection and no prior radical cure PQ treatment, were recruited into the clinical study and completed the PK sampling.^9^ PQ (0.5 mg base/kg) was given to the women (nonfasted state) once daily for 14 days after the breastfeeding infants were at least 28 days old. The exclusively breastfed infants were aged 1.5–22 months (average 5.0) and 33% were girls. PK samples included maternal venous and capillary blood, breast milk, and infant capillary blood samples.

An independent clinical study was used to verify the PBPK model for PQ in children prior to running simulations in breastfeeding infants. A PK study was conducted in 15 healthy G6PD‐normal Papua New Guinean children, aged 6–10 years (40% girls), following administration of a single dose of 0.5 mg/kg in a fed state.^17^ Venous blood samples were drawn during a 168‐h period postdose.

In a separate clinical study conducted in Brazil,^18^ 22 children aged 2–3 years, 20 aged 4–8 years, 21 aged 9–11 years, and 22 aged 12–14 years received daily doses of 0.42 mg/kg, 0.5 mg/kg, 0.44 mg/kg, and 0.46 mg/kg PQ, respectively, for 7 days for treatment of *P. vivax* malaria. Blood samples were collected over the range 2–3.5 h (median 2.5 h).

### Virtual populations

The clinical lactation study was conducted in clinics along the Thailand–Myanmar border; thus, subjects mainly consisted of workers and refugees from Myanmar, predominantly from the Burman or Karen ethnic groups. As a representative virtual population was not available, all simulations were conducted using a North European (NE) White group as the baseline population. Default parameter values for creating this NE White population (physiological parameters, including liver volume, blood flows, and enzyme abundances) have been described previously.^19^

The pediatric module within the Simcyp Simulator allows prediction of PKs in neonates and infants. A full PBPK model together with extensive libraries on pediatric demography (age, height, weight, and body surface area), developmental physiology (liver size, renal function, and liver blood flow), and biochemistry (albumin, AAG [α1‐acid glycoprotein], and CYP ontogeny) is integrated and the underlying algorithms describing these changes are described in detail elsewhere.^20^, ^21^ The default pediatric population is also based on a White population. Body height is typically expressed as a polynomial function of age, whereas body weight is described as a function of both age and height. The coefficients for these equations as well as the coefficient of variation of height and weight were determined by fitting them to observed pediatric data from UK growth charts separately for boys and girls. To generate children with heights and weights of virtual subjects consistent with those from the actual pediatric clinical studies,^17^ the constants in the equations were changes and integrated within the Simcyp Simulator via a Lua script.

#### Height

Boys less than 18 years old:

(1) $$\begin{array}{c} \text{Height}\;\left(\mathrm{cm}\right)=0.0000176179*{\mathrm{age}}^{7}-0.00119874*{\mathrm{age}}^{6}\\ \;+0.0323848*{\mathrm{age}}^{5}-0.444112*{\mathrm{age}}^{4}\\ \;+3.2946*{\mathrm{age}}^{3}-13.2191*{\mathrm{age}}^{2}\\ \;+33.75*\mathrm{age}+45.62152 \end{array}$$

Girls less than 18 years old:

(2) $$\begin{array}{c} \text{Height}\;\left(\mathrm{cm}\right)=-0.00000151027*{\mathrm{age}}^{8}+0.000121261*{\mathrm{age}}^{7}\\ \;-0.0040023*{\mathrm{age}}^{6}+0.070179*{\mathrm{age}}^{5}\\ \;-0.708233*{\mathrm{age}}^{4}+4.1872*{\mathrm{age}}^{3}\\ \;-14.3393*{\mathrm{age}}^{2}+33.84778*\mathrm{age}+40.535477 \end{array}$$

#### Weight

Boys less than 18 years old:

(3) $$\begin{array}{c} \text{Weight}\;\left(\mathrm{kg}\right)=6.026*\left(1.0-\text{math.exp}\;\left(\mathrm{age}*-1.2\right)\right)\\ \;+\text{math.exp}\;\left(\left(\text{height}*0.0209\right)+\left(0.023*\mathrm{age}\right)\right) \end{array}$$

Girls less than 18 years old:

(4) $$\begin{array}{c} \text{Weight}\;\left(\mathrm{kg}\right)=4.054*\;\left(1.0-\text{math.exp}\;\left(\mathrm{age}*-1.57\right)\right)\\ \;+\text{math.exp}\;\left(\left(\text{height}*\;0.0224\right)+\left(0.019*\;\mathrm{age}\right)\right) \end{array}$$

### Simulations

Where possible, the demographic (including age, gender, weight, and height) characteristics and dose regimens used in the simulations were matched to those of subjects recruited into the clinical studies. In addition, the number of virtual subjects was based on 10 trials of the number of subjects used in the corresponding clinical study.

Thus, 10 virtual trials of 20 women aged 18–40 years receiving multiple oral doses of PQ (0.5 mg/kg q.d. for 14 days) were generated and the simulated and observed^9^ concentrations and PKs of PQ in plasma and milk were compared. Simulations were run using the observed milk‐to‐plasma (M/P) ratio of 0.34.

Algorithms for predicting the PQ M/P ratio have been described in detail previously.^11^ Key parameters include the physicochemical parameters of the drug and the fat content and pH of the milk.^22^ As the M/P ratio affects the IDD, sensitivity analyses were performed to assess the effects of temporal changes in fat content and pH on the predicted M/P ratio.

Ten virtual trials of 15 children aged 6 to 10 years (40% girls) receiving a single oral dose of 0.5 mg/kg PQ were generated and the simulated and observed^17^ concentrations of PQ in plasma were compared.

Ten virtual trials of 22 children (50% girls) aged 2 to 3 years, 20 aged 4–8 years, 21 aged 9–11 years, and 22 aged 12–14 years receiving daily doses of 0.42 mg/kg, 0.50 mg/kg, 0.44 mg/kg, and 0.46 mg/kg PQ, respectively, were generated and the simulated and observed^18^ concentrations of PQ in plasma were compared.

In the clinical lactation study,^9^ IDDs were estimated from the milk exposures of PQ.^9^ The total cumulative PQ dose expected to be consumed by the infant during breastfeeding over the 14‐day course was ~0.042 mg/kg, corresponding to 2.98 μg/kg/day. The highest cumulative infant dose was estimated at 0.127 mg/kg, or 9.07 μg/kg/day. As these values were higher than the IDD based on a time‐averaged milk concentration, they represented the worst‐case scenario and were also applied in simulations.

Ten virtual trials of 20 infants (33% girls) aged 1.5–22 months (0.125–1.83 years), consistent with the clinical study, receiving the IDD were generated. The time‐varying physiology features^14^ were used to simulate PKs in infants. It should be noted that in the clinical study, the infant feeding times were not controlled or reported. Thus, it was assumed that the IDD was consumed across six feeding times (4 h apart).

Simulations were repeated in neonates aged up to 28 days. A default ontogeny for CYP2D6 based on literature data is available within the Simcyp Simulator and was applied in simulations.^19^ No ontogeny data are available for MAO‐A. Given that the contribution of the enzyme to PQ metabolism is about 90% in adults, various scenarios were assessed; a slow and no ontogeny was applied to the MAO‐A pathway in the simulations. AAG is the main binding protein for PQ.^23^ As it is an acute phase reactive protein, its level can increase up to four‐fold in children with malaria and severe malnutrition.^24^ Thus, a sensitivity analysis was conducted in infants and neonates less than 28 days old to assess the effects of a four‐fold increase in AAG on the PQ exposure.

## RESULTS

### Predicted versus observed PQ plasma and milk concentration time profiles in nursing mothers

**FIGURE 1:**
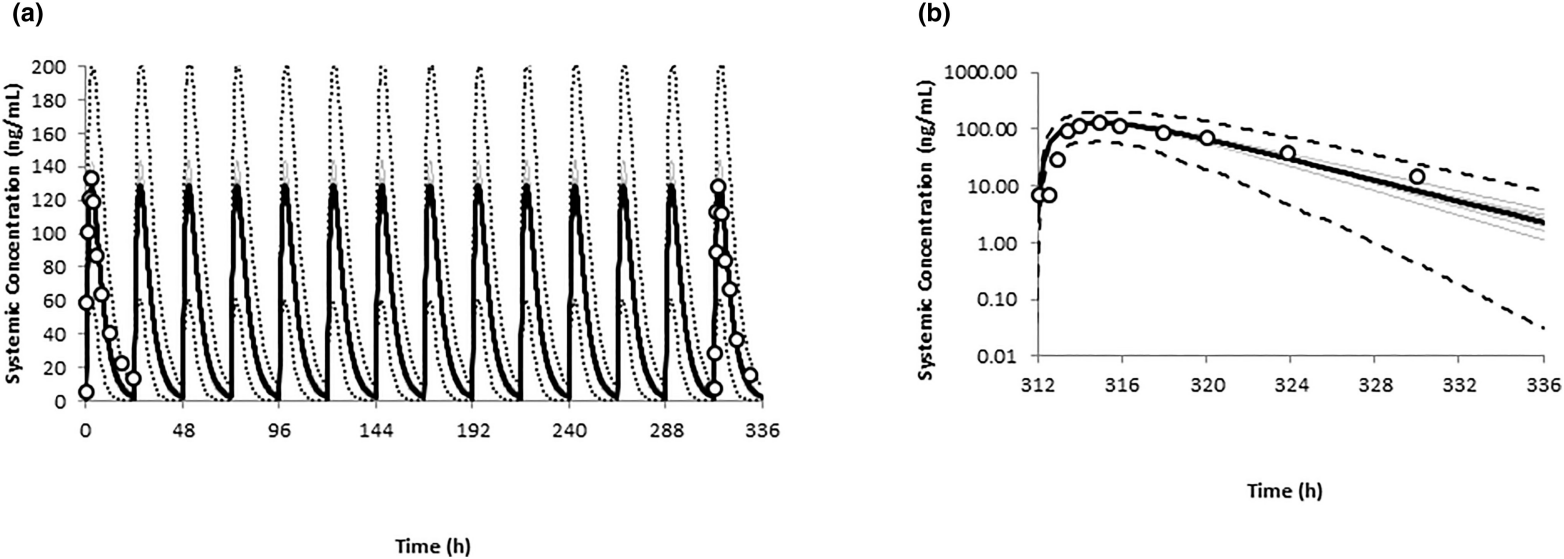
Simulated and observed PQ plasma concentration profiles in women at postpartum. Simulated (black line) and observed (data points) mean concentration time profiles of PQ following 0.5 mg/kg daily doses in nursing mothers (a) and on the last day of dosing (b). The dashed black lines represent the 5th and 95th percentiles of the total virtual population and the gray lines represent the individual trials. PQ, primaquine.

**FIGURE 2:**
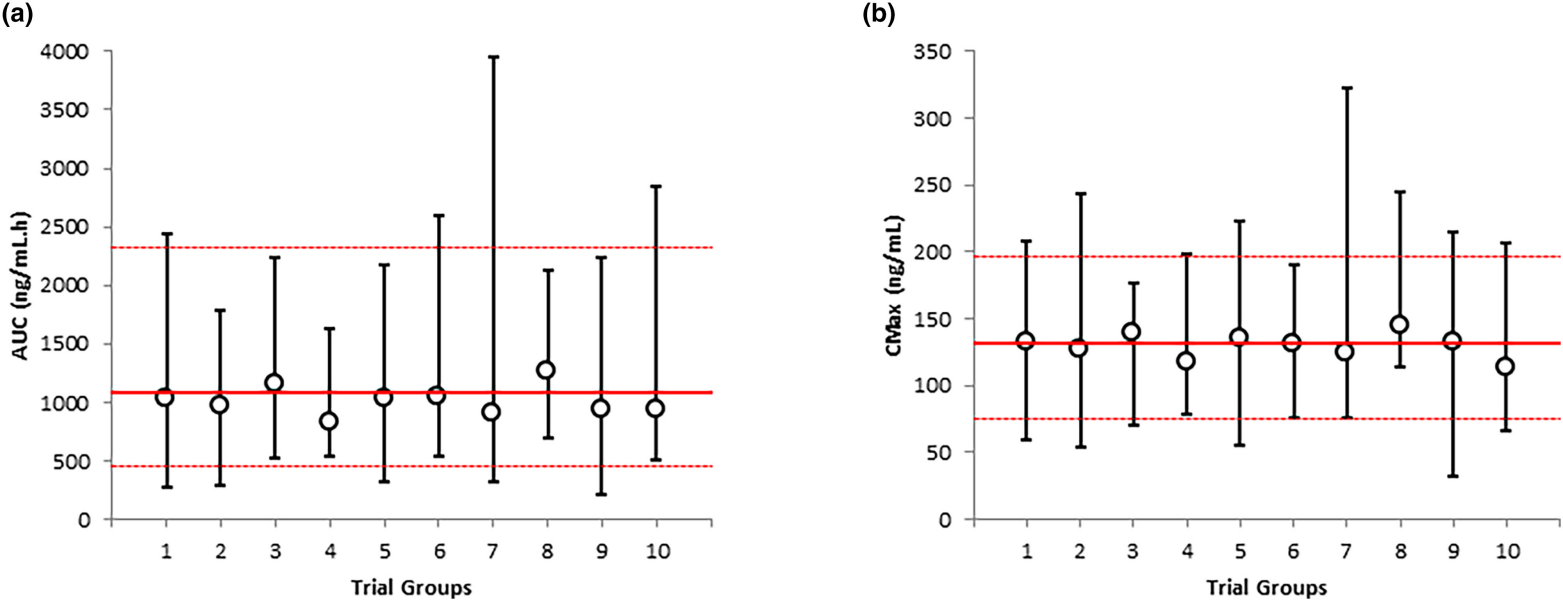
Simulated and observed variability in PQ plasma concentrations in women at postpartum. Simulated (symbols and black lines) and observed (red lines) variability in plasma exposure of PQ after 0.5 mg/kg daily dosing of PQ in nursing mothers. Median and range are shown for AUC (a) and *C*_max_ (b) for each virtual trial and the observed data. AUC, area under the curve; *C*_max_, maximum plasma concentration; PQ, primaquine.

The simulated and observed mean plasma PQ concentration time profiles following once daily dosing of 0.5 mg/kg PQ in nursing mothers are shown in Figure 1. Predicted median maximum plasma concentration (*C*_max_) and area under the curve (AUC) values of 129 ng/mL and 988 ng/mL*h on day 14 were consistent with observed values of 132 ng/mL and 1090 ng/mL*h, respectively. Furthermore, except for three trials, the predicted median and range of *C*_max_ and AUC values for the 10 trials of 20 virtual subjects captured the observed variability in PQ exposure (Figure 2).

The predicted M/P ratio was 0.47 compared with the observed value of 0.34. The sensitivity analyses of fat content (2%–5%) and pH of breastmilk on M/P ratio, which can affect the IDD, were performed. The predicted M/P ratio was relatively insensitive to changes in fat content (2%–5%) of breast milk with values ranging from 0.48 to 0.47. The predicted M/P ratio increased from 0.2 to 0.47 with the change of breast milk pH from 7.6 in colostrum to 7.2 in mature milk.

### Predicted versus observed PQ plasma concentration time profiles in children

**FIGURE 3:**
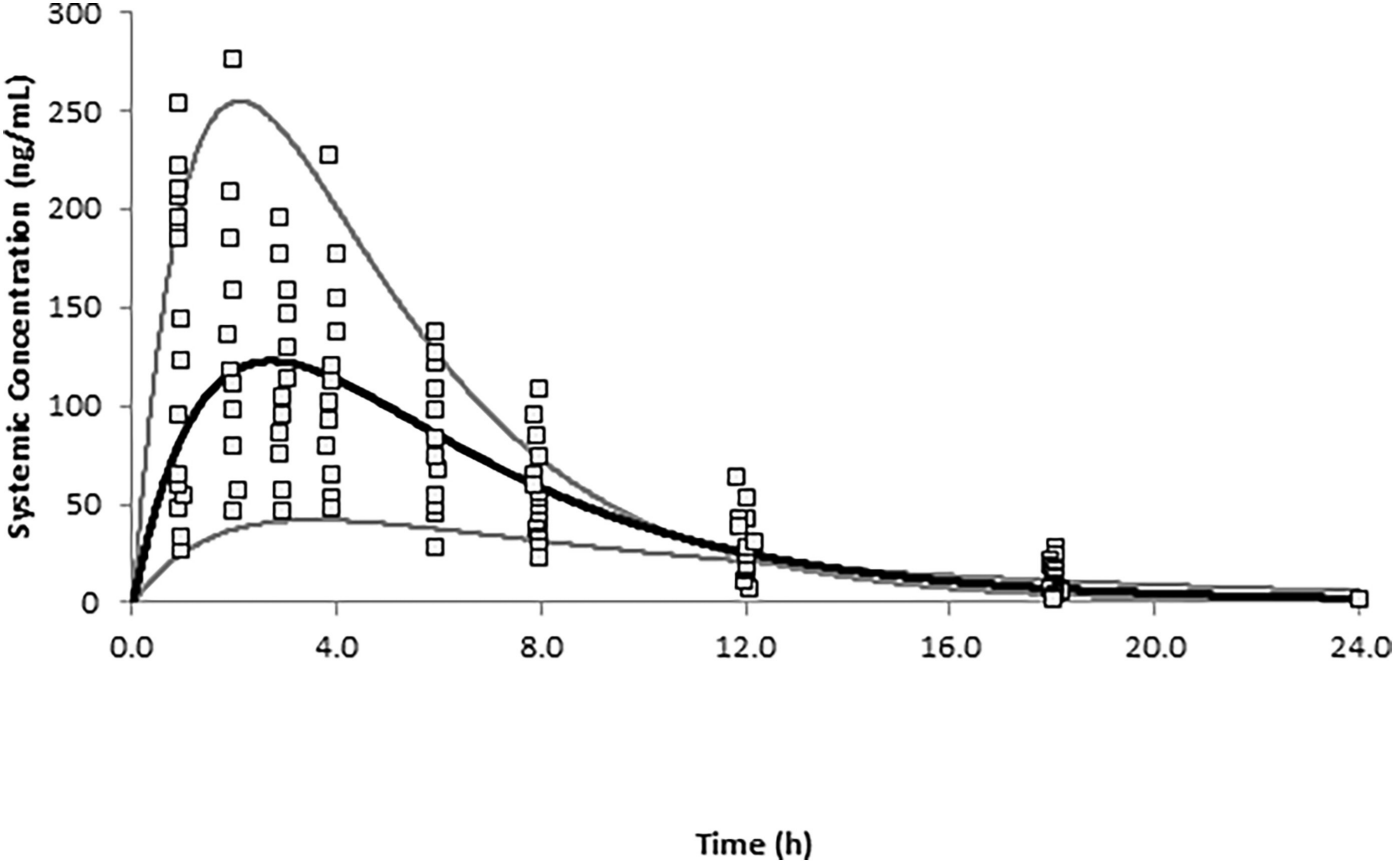
Simulated and observed PQ plasma concentration profiles in children. Simulated (black line) and observed (data points) mean concentration time profiles of PQ following 0.5 mg/kg daily doses in children aged 6–10 years. The gray lines represent the minimum and maximum profiles of the total virtual population. PQ, primaquine.

The simulated mean plasma PQ concentration time profile following a single oral dose of PQ (0.5 mg/kg) in children aged 6 to 10 years is shown in Figure 3. Visual predictive checks indicate that most of the observed data fall within the 5th and 95th percentiles of the simulated population confirming that the PBPK model can capture the variability in children within this age range. Although observed PK parameters were not available for direct comparison, it is interesting to note that the predicted exposures in children are similar to those in adults, a finding that is consistent with the clinical study.^17^

Predicted *C*_max_ values on the last day of 7 days of dosing in children aged 2–3 years, 4–8 years, 9–11 years, and 12–14 years receiving daily doses of 0.42 mg/kg, 0.5 mg/kg, 0.44 mg/kg, and 0.46 mg/kg PQ, respectively, were 113, 127, 106, and 112 ng/mL, respectively. Whereas observed PK parameters were not available for direct comparison, observed PQ concentrations in blood samples collected over the range 2–3.5 h (median 2.5 h) postdose on day 7 were 90.5, 94, 93, and 103 ng/mL, respectively.

### Using the clinical lactation data to predict PQ plasma concentrations in infants greater than 28 days old

**FIGURE 4:**
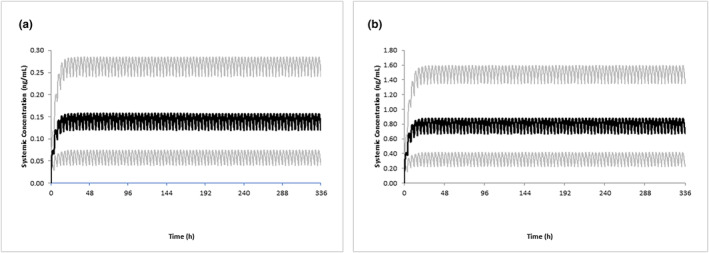
Simulated and observed PQ plasma concentration profiles in breastfeeding infants. Simulated mean concentration time profiles of PQ following multiple oral daily doses of 2.98 μg/kg (a) and 9.07 μg/kg (b) in children aged greater than or equal to 28 days old. The gray lines represent the 5th and 95th percentiles of the total virtual population. It was assumed that the total daily dose was split across six doses given every 4 h. PQ, primaquine.

**FIGURE 5:**
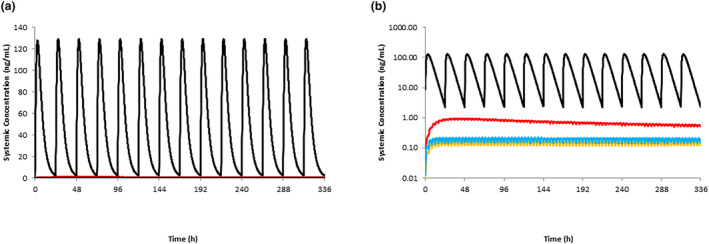
Simulated PQ plasma concentration profiles in breastfeeding infants relative to mothers. Simulated mean concentration time profiles of PQ following 0.5 mg/kg daily doses in nursing mothers (black line) and oral daily doses of 2.98 μg/kg in children aged greater than or equal to 28 days (orange), in children aged less than 28 days assuming no ontogeny for MAO‐A (blue) and a slow ontogeny (red). It was assumed that the total daily dose was split across six doses given every 4 h. Linear (a) and log‐linear (b) plots are shown. MAO‐A, monoamine oxidase A; PQ, primaquine.

Using the reported average IDD of 2.98 μg/kg and assuming six feedings per day (0.497 μg/kg), predicted mean *C*_max_ time profiles in infants aged 1.5–22 months are shown in Figure 4. The variability in PQ exposure across the virtual population is also shown (Figure 4). All predicted infant PQ concentrations were relatively low (~1000‐fold lower) compared to the mothers; median *C*_max_ values were 0.14 ng/mL versus 132 ng/mL, respectively (Figure 5). This remained so, even when the highest observed IDD was used in simulations (9.07 μg/kg; Figure 4). Although no observed data were available for comparison, as all PQ concentrations in the infant plasma were below the lower limit of quantitation (LLOQ) of 1–2 ng/mL (except one sample of 2.59 ng/mL on day 7), the PBPK predicted infant concentrations appeared reasonable and were also found to be below the LLOQ.

A sensitivity analysis to assess the effects of a four‐fold increase in AAG in infants indicated that the ratio of *C*_max_ in infants to mothers increased from 0.12% to 0.35% over the four‐fold range for the reported average IDD and from 0.67% to 1.96% for the highest reported IDD.

### Using the clinical lactation data to predict PQ plasma concentrations in neonates less than 28 days old

Using the reported average IDD of 2.98 μg/kg and assuming six feedings per day (0.497 μg/kg), predicted mean *C*_max_ time profiles in infants aged less than 28 days are shown in Figure 5. In the absence of any information defining the ontogeny of MAO‐A, the effects of applying no ontogeny for MAO‐A and a slow ontogeny were also investigated. Even when a slow ontogeny was assumed (the most conservative assessment), PQ exposures remained relatively low (100‐fold lower) compared to exposures in the mother (Figures 4 and 5).

A sensitivity analysis to assess the effects of a four‐fold increase in AAG in neonates indicated that the ratio of *C*_max_ in infants to mothers increased from 0.16% to 0.44% over the four‐fold range when no ontogeny was applied and did not change from 0.44% assuming the slow ontogeny.

## DISCUSSION

A PBPK model for PQ reported previously^16^ was applied in this study to predict the exposure of PQ in mothers and in breastfeeding infants. As the original PQ model was developed in adults, simulations were also conducted to verify the performance of the model in children using available clinical data. Plasma exposures following oral daily dosing of 0.5 mg/kg in the nursing mothers were accurately captured (within 1.1‐fold), including the observed ranges.^9^ Simulations of children aged 6 to 10 years receiving a single oral dose of 0.5 mg/kg in an independent clinical study indicated that the predicted exposures (including variability) were entirely consistent with the observed data^17^ (Figure 3). Furthermore, in children aged to 2–3 years old, predicted PQ exposures were within 1.25‐fold of observed data.^18^ Although a White population was used in the simulations, ensuring that age, gender, and weight (when possible) of the virtual subjects matched those of the individuals from the different ethnic groups recruited into the clinical studies, allowed accurate recovery of the observed data.

Reported IDDs based on milk data were used to predict the exposure of PQ in breastfeeding infants greater than or equal to 28 days. As the corresponding PQ concentrations in the clinical study were below measurement thresholds (1–2 ng/mL), it was not possible to directly compare the exposures.^9^ However, it should be noted that the simulations indicate that all PQ concentrations in infants were less than or equal to 2 ng/mL. On average, the predicted *C*_max_ and AUC of PQ in breastfeeding infants are less than 0.13% of the mothers (about a 1000‐fold lower). Furthermore, in simulations involving neonates less than 28 days old, PQ exposures remain less than 0.16% of the mothers. There are no published data regarding the ontogeny of MAO‐A, which is the main enzyme that contributes to PQ metabolism (about 90% in adults). However, even assuming that MAO‐A increases slowly with age, the predicted relative exposure of PQ remains low in neonates (<0.46%).

It should be noted that in our study it was assumed that the same IDD of 2.98 μg/kg was consumed by the breastfeeding infants irrespective of age. This IDD estimate taken from the clinical lactation study^7^ was based on a milk consumption of 150 mL/kg/day. According to the FDA guidance, it is stated that while assuming this intake is reasonable for estimation of the IDD, greater volumes may be consumed in early infancy and additional consideration should be given to estimates of infant risk based on a 200 mL/kg/day milk intake.^25^ Even in this case (using 200 vs. 150 mL/kg/day), as the PKs of PQ is relatively linear, the higher IDD (4/3 × higher) would result in exposures that remained relatively low in neonates.

As the IDD is estimated from milk data, it is dependent on factors affecting the passage of drugs into the milk.^26^, ^27^ Almost all drugs pass into milk from maternal plasma by passive diffusion; the M/P ratio is affected by the composition of the milk (aqueous, lipid, protein, and pH) and the physicochemical characteristics of the drug (protein binding, lipophilicity, and pKa). The composition of a mother's breast milk changes significantly during the first 2 months postpartum, adapting itself to the evolving needs of the growing newborn.^22^ Colostrum is the first fluid that is expressed by the mother after delivery and its primary function appears to immunological rather than nutritional. Hence, the fat content in breast milk is typically low (about 2.0%) soon after the birth of the infant before increasing over the next 6 months or so. In general, human breast milk fat content ranges from 3.5% to 4.5%. At about 4% (default setting in lactating women), the predicted M/P ratio is 0.47, which is reasonably consistent with the observed value of 0.34. The physicochemical characteristics of PQ are such that the M/P ratio is not sensitive to changes in fat content (2%–5%) with predicted values ranging from 0.48 to 0.47. The pH of breast milk also changes during the first month postpartum, from pH of 7.6 in colostrum (1–4 days postpartum), to 7.44 in transitional milk (5–30 days postpartum) and 7.2 in mature milk (>30 days postpartum).^22^ These changes lead to an increase in the M/P ratio from 0.2 (pH 7.6) to 0.47 (pH 7.2).

Another factor that needs to be considered is how the plasma protein binding of PQ to AAG changes in mothers and children. During pregnancy, AAG levels decrease to 0.6 g/L but return to the non‐pregnancy levels of 0.7 g/L postpartum.^23^ Thus, the change in the unbound fraction (0.33 to 0.29) is not likely to affect the milk concentration of PQ significantly or indeed the M/P ratio (and the IDD) postpartum and during breastfeeding. Furthermore, as AAG is an acute phase reactive protein, its level can be increased by four‐fold in children with malaria and severe malnutrition.^24^ Given the ontogeny associated with AAG over this age range and potential changes due to disease, there could be significant changes in the unbound fraction of PQ which could affect the clearance and hence the exposure. Based on the average reported IDD, the effects of the four‐fold increase in AAG was to increase the relative exposure from 0.12% to 0.35% in infants compared to mothers whereas remaining the same in neonates less than 28 days old assuming a slow ontogeny (0.44%).

The approach described here demonstrates how PBPK modeling can be used to predict drug exposures in both nursing mothers and infants while accounting for complex factors, such as time varying physiology.^12^, ^13^, ^28^ Although it is accepted that this is an emerging area of interest with significant clinical relevance, evaluation of such approaches is ongoing and the results are promising. PBPK modeling can be used to increase the utility and efficiency of lactation studies with the goal of providing important information to prescribers and their breastfeeding patients.^29^ For example, PBPK can be used to fill the gaps in clinical studies, such as in the first month postpartum when recruitment into clinical lactation studies can be difficult or to predict infant exposure when “milk‐only” or “milk and maternal plasma” study designs are used. Currently, PQ is contraindicated in lactating women unless G6PD information is available in infants.^30^, ^31^, ^32^ As G6PD testing may not be readily available and is quite invasive, the WHO recommends delaying treatment in mothers until their nursing infants are at least 6 months old and determined to be G6PD normal. In a recent clinical study,^30^ it was reported that single low‐dose PQ (median – 0.21 mg/kg; range – 0.07 mg/kg to 0.4 mg/kg) was well‐tolerated in 530 children aged 6 months to 11 years. This large, multicenter, placebo‐controlled study whose primary outcome was the development of acute anemia included many children with a genotypically confirmed G6PD deficiency. Furthermore, the safety profile of this treatment was found to be similar to that of the placebo. In the clinical lactation study and our simulations, 2.98 μg/kg was “consumed” daily over 14 days (0.042 mg/kg in total). This five‐fold lower dose taken over 14 days is highly unlikely to cause hemolysis in infants, even those who are G6PD‐deficient.

In summary, our PBPK model published previously for PQ was used to predict exposures in breastfeeding infants as a consequence of PQ dosing (0.5 mg/kg) in mothers; on average, the predicted exposures of PQ in infants are less than 0.13% of the mothers, even with the most conservative assumptions around enzyme and plasma protein ontogenies. Thus, the findings of our study support the recommendation made by the authors who reported the results of the clinical study,^9^ that is, that PQ should not be withheld in lactating women as it is unlikely to cause adverse events in breastfeeding infants greater than or equal to 28 days old.

## AUTHOR CONTRIBUTIONS

K.R.Y. and X.P. wrote the manuscript. K.R.Y., X.P., K.A., A.P., and L.M.A. designed the research. K.R.Y., X.P., K.A., and A.P. performed the research. K.R.Y., X.P., K.A., and A.P. analyzed the data.

## FUNDING INFORMATION

This work was funded by the Bill and Melinda Gates Foundation (INV‐040110). The views expressed in this work do not reflect official views of the Bill & Melinda Gates Foundation.

## CONFLICT OF INTEREST STATEMENT

All author are employees of Certara UK Limited (Simcyp Division) and may hold shares in Certara. As Deputy Editor in Chief of CPT: Pharmacometrics & Systems Pharmacology, Karen Rowland Yeo was not involved in the review or decision process for this paper.
